# Integrative Analysis of Cell Crosstalk within Follicular Lymphoma Cell Niche: Towards a Definition of the FL Supportive Synapse

**DOI:** 10.3390/cancers12102865

**Published:** 2020-10-05

**Authors:** Céline Pangault, Patricia Amé-Thomas, Delphine Rossille, Joëlle Dulong, Gersende Caron, Céline Nonn, Fabrice Chatonnet, Fabienne Desmots, Vincent Launay, Thierry Lamy, Thierry Fest, Karin Tarte

**Affiliations:** 1 UMR_S 1236, Univ Rennes, INSERM, Établissement Français du Sang (EFS) Bretagne, LabEx IGO, F-35000 Rennes, France; celine.pangault@univ-rennes1.fr (C.P.); patricia.ame@univ-rennes1.fr (P.A.-T.); delphine.rossille@univ-rennes1.fr (D.R.); joelle.dulong@univ-rennes1.fr (J.D.); gersende.lacombe@univ-rennes1.fr (G.C.); celine.nonn@univ-rennes1.fr (C.N.); fabrice.chatonnet@univ-rennes1.fr (F.C.); fabienne.desmots-loyer@univ-rennes1.fr (F.D.); thierry.lamy@univ-rennes1.fr (T.L.); 2 Laboratoire Hématologie, Centre Hospitalier Universitaire de Rennes, F-35000 Rennes, France; 3 Laboratoire Immunologie, Centre Hospitalier Universitaire de Rennes, F-35000 Rennes, France; 4 Laboratoire Suivi Immunologique des Thérapeutiques Innovantes (SITI), Centre Hospitalier Universitaire de Rennes, F-35000 Rennes, France; 5 Service Hématologie Clinique, Centre Hospitalier Yves Le Fol, F-22000 Saint Brieuc, France; vincent.launay@ch-stbrieuc.fr; 6 Service Hématologie Clinique, Centre Hospitalier Universitaire de Rennes, F-35000 Rennes, France

**Keywords:** follicular lymphoma, B cells, Tfh, mesenchymal stromal cells

## Abstract

**Simple Summary:**

Follicular lymphoma, the most frequent indolent non-Hodgkin’s B cell lymphoma, arises from a germinal center B cell proliferation supported by a multidirectional crosstalk with the tumor microenvironment, in particular with follicular helper T cells and mesenchymal stromal cells. Here, we explored this complex network, starting from a comparative analysis of the molecular signatures of B cells, T cells, and stromal cells obtained from normal versus lymphoma tissues, and focusing on deregulated genes reflecting the crosstalk between these three cell subsets organizing the lymphoma cell niche. This helps us to point out new lymphoma-specific pathways, related to transcriptomic and functional specific features of T and stromal cells, and contributing to tumor B cell support directly or through the recruitment and/or activation of other pro-tumoral cell components. In the future, targeting these cell interactions with specific drugs in the FL niche could represent an attractive option for novel therapeutic strategies.

**Abstract:**

Follicular lymphoma (FL), the most frequent indolent non-Hodgkin’s B cell lymphoma, is considered as a prototypical centrocyte-derived lymphoma, dependent on a specific microenvironment mimicking the normal germinal center (GC). In agreement, several FL genetic alterations affect the crosstalk between malignant B cells and surrounding cells, including stromal cells and follicular helper T cells (Tfh). In our study, we sought to deconvolute this complex FL supportive synapse by comparing the transcriptomic profiles of GC B cells, Tfh, and stromal cells, isolated from normal versus FL tissues, in order to identify tumor-specific pathways. In particular, we highlighted a high expression of *IL-6* and *IL-7* in FL B cells that could favor the activation of FL Tfh overexpressing IFNG, able in turn to stimulate FL B cells without triggering MHC (major histocompatibility) class II expression. Moreover, the glycoprotein clusterin was found up-regulated in FL stromal cells and could promote FL B cell adhesion. Finally, besides its expression on Tfh, CD200 was found overexpressed on tumor B cells and could contribute to the induction of the immunosuppressive enzyme indoleamine-2,3 dioxygenase by CD200R-expressing dendritic cells. Altogether our findings led us to outline the contribution of major signals provided by the FL microenvironment and their interactions with malignant FL B cells.

## 1. Introduction

Germinal centers (GC) are transient and dynamic specific structures within lymph nodes (LN) that are dedicated to B cell selection and differentiation into high-affinity antibody-secreting cells [1,2]. Physiological GC reaction is initiated after a cognate interaction between an antigen-specific B cell and a pre-activated CD4^pos^ T cell at the T/B border. After undergoing rapid cell proliferation and somatic hypermutations of immunoglobulin (Ig)-variable regions in the GC dark zone, GC B cells (BGC) migrate to the light zone, and are named centrocytes. Non-proliferative centrocytes are then subjected to affinity-based selection driven by antigen-presenting follicular dendritic cells (FDC) and T follicular helper cells (Tfh), allowing them to either undergo additional rounds of somatic hypermutations and selection, or to terminally differentiate into memory B cells or Ig-secreting plasma cells. The GC B cell reaction relies on a finely-balanced gene network involving two groups of transcription factors: (i) genes maintaining the B cell fate, including *PAX5*, *BCL6* and *BACH2*, and (ii) genes required for antibody-secreting cell differentiation, including *IRF4*, *BLIMP1*, and *XBP1*. B cell maturation within GC depends on dynamic interactions between B cells, Tfh, and FDC, and leads to genome modifications favoring unwanted alterations. In agreement, whereas up to 50% of GC B cells undergo programmed cell death every 6 h due to low-affinity Ig expression and/or genome alterations [3], the GC reaction is prone to be hijacked by oncogenic processes leading to malignant B cell transformation.

Follicular lymphoma (FL) is the most frequent indolent non-Hodgkin’s B cell lymphoma (NHL-B) and is considered as a prototypical centrocyte-derived lymphoma. The largest majority of FL cases harbor a *BCL2/IGH* translocation arising during the VDJ rearrangement process in the bone marrow (BM). Nevertheless, this translocation, which allows the overexpression of the anti-apoptotic molecule BCL2, could be detected at low frequency within recirculating post-GC memory B cells of most healthy individuals, indicating that it is not sufficient to trigger overt FL [4]. Advances in high-throughput genetic analyses have revealed the complex landscape of additional molecular events that support FL development [5,6,7]. Of note, beyond the well-accepted identification of FL B cells as centrocytes that fail to differentiate [8], recent single-cell transcriptomic analyses revealed a desynchronization of the GC-specific gene expression program in FL malignant cells that might adopt new dynamic modes of functional diversity [9]. Moreover, studies interrogating sequential FL biopsies revealed that FL does not arise through a linear evolutionary pattern and that an underestimated degree of spatial or intra-tumor heterogeneity exists [10]. Interestingly, some recurrent genetic events act through the modulation of the crosstalk between FL B cells and surrounding cells of their microenvironment. As an example, we demonstrated that the introduction of *n*-glycosylation acceptor sites harboring unusual high-mannose oligosaccharides in FL BCR triggers the interaction of malignant B cells with DC-SIGN-expressing tumor-infiltrating macrophages resulting in BCR-dependent FL B cell activation [11]. In addition, malignant FL B cells display a substantial dependency on their microenvironment organized as specific supportive cell niches providing survival and proliferation signals (reviewed in [12,13]). Composition and spatial organization of the FL niches have a huge impact on patient prognosis, as initially described by Dave et al. [14]. FL neoplastic nodules typically retain the main features of non-malignant GC, and ectopic lymphoid structures are induced within FL-invaded BM, displaying lymphoid-like stromal cells and CD4^pos^ T cells [15,16]. These two cell types are required in FL cell niches and both display phenotypic, transcriptomic and functional specific features contributing to tumor B cell growth and disease progression either directly or through the recruitment and/or activation of other pro-tumoral cell components [16,17,18]. Overall, the network of interactions between FL B cells, stromal cells, and Tfh, referred herein as FL supportive synapse, mediates the education of the FL tumor microenvironment. Although recently published data provide new insights into the biology of these three partners in the FL context, their crosstalk remains poorly explored.

In this study, we sought to deconvolute the crosstalk between FL B cells, Tfh, and stromal cells. To this end, we compared the transcriptome of cell populations isolated from non-malignant tissues versus FL biopsies, and identified tumor-related pathways involving cytokines, adhesion molecules, and their receptors. Concerning FL Tfh, we found an up-regulation of *IL-4*, *IFNG*, *TNFA*, and *IL-2*, associated with a cell activation signature, which paralleled the concomitant high expression levels of *IL-6* and *IL-7* in FL B cells. In addition, we demonstrated that FL B cells, although possessing a functional IFN-γ pathway, were not able to positively regulate HLA-DR expression. We also described an elevated expression of CD200 in FL supportive synapse, triggering the expression of the immunosuppressive indoleamine-2,3 dioxygenase (IDO) enzyme by CD200R-expressing dendritic cells (DC). Finally, our data highlighted an elevated expression of *CLU* by FL stromal cells, and highlighted that clusterin could mediate FL B cell adhesion, thus potentially contributing to FL dissemination.

## 2. Results

### 2.1. Global Analysis of Molecular Connections at FL Synapse

The central aim of our study was to establish a comprehensive characterization, based on the transcriptome exploration, of the interactions between the three main actors of the FL tumor, namely tumor B cells, Tfh, and stromal cells. Based on our previous works, we focused our analyses on cells isolated from FL biopsies and non-malignant samples, which together represented six (3 nonmalignant and 3 FL-derived) different populations and forty-seven samples (Table 1). B and Tfh cells were purified using fluorescent-activated cell sorting, while stromal cells were isolated after culture of BM samples issued from healthy donors and FL patients with invaded BM. The BM-derived mesenchymal stromal cells (MSC) obtained from FL patients have been previously shown as a valuable model for studying FL-stromal reprogramming [19]. A quick data analysis of the gene expression profiles (GEP) confirmed the high expression of the expected cell-specific markers, including *CD19* and *CD40* for B cells, *CD3*, *CD4*, *CD40L* for Tfh, and *CD90/THY1*, *CD105/ENDOG*, *CD106/VCAM1* for MSC, without detectable cross-contamination.

**Figure 1:**
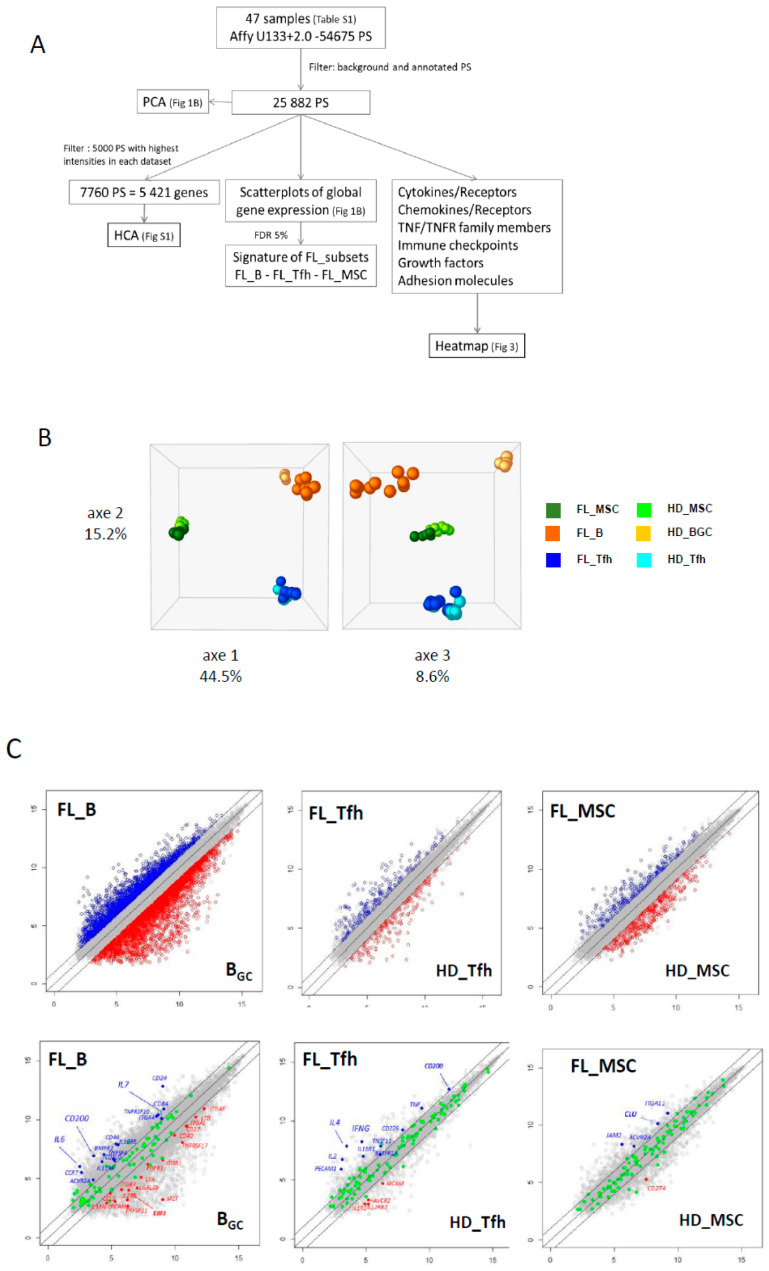
(**A**) Microarray data analysis strategy. (**B**) Principal component analysis (PCA) shows tight clustering of the mesenchymal stromal cell (MSC) compartment, B (B) and T follicular helper (Tfh) lymphocytes from normal and tumoral contexts. The first three axes total 68.3% of the total inertia of the dataset. (**C**) Scatterplots of pairwise global gene expression comparison between FL (follicular lymphoma) and non-tumoral samples, in B lymphocytes (left panel), Tfh (middle panel) or stromal cells (right panel). Gene expression values are plotted on a log scale. Genes that were differentially expressed (FDR (false discovery rate) ≤ 5%) with a fold change ≥2 (logFC ≥ 1) are indicated in red and blue. Top panel: representation of all annotated genes (21,319 PS (probesets) in B lymphocytes, 19,859 PS in Tfh and 20,142 PS in stromal cells). Bottom panel: highlight of genes (in green) differentially expressed (FDR ≤ 5%) with a fold change ≥2 (logFC ≥ 1).

We selected a multistep process for our dataset analysis (Figure 1A). In the first step, we reduced our dataset to the 25,882 expressed probesets (PS), corresponding to 13,918 annotated genes. This large-scale approach allowed us to draw the global landscape of the GEP in normal and FL contexts. PCA analysis confirmed tight clustering of each cell type, with the first two axes sustaining nearly 60% of the statistical variance, separating B cells, Tfh, and MSC regardless of their origin (Figure 1B). The third axis (8.6% of the variance) allowed us to segregate FL B cells from their normal GC B cell counterparts, confirming a distinct molecular FL-B signature, while Tfh and MSC were not separated depending on the normal or FL contexts. These data were confirmed by unsupervised hierarchical clustering performed on the 5000 PS with the highest intensities for each population (Figure S1).

**Figure 2:**
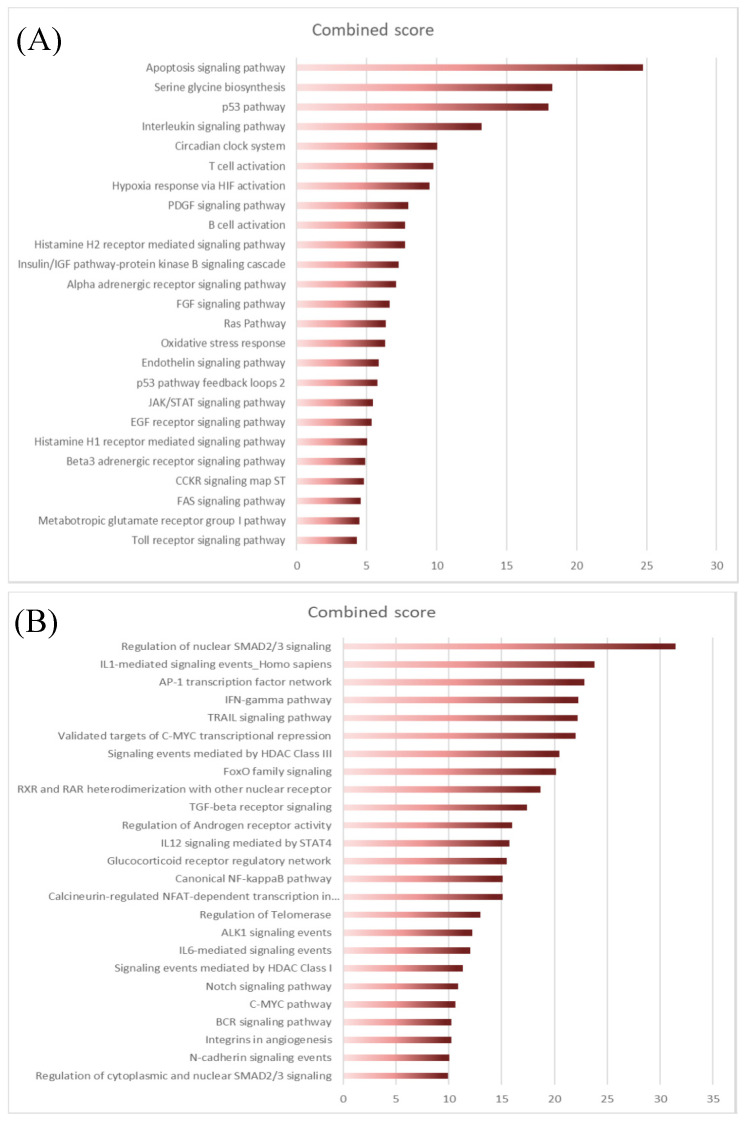
EnrichR analysis of differentially expressed transcriptome of FL niche players using NCI Nature library (**A**) or Panther library (**B**). The top 25 pathways were sorted according to the EnrichR combined scores.

The next step was to explore the differentially expressed genes (DEG) between normal and FL settings. By performing moderated t-tests with FDR correction (FDR < 5%), we identified, respectively, 616 (281 up and 355 down) and 1193 (383 up and 887 down) DEG in FL-Tfh and FL-BM-MSC, compared to their normal counterparts. As expected, the number of DEG was higher for B cells with 2998 up- and 3342 down-regulated genes in FL-B cells compared to normal GC B cells (Figure 1C). Based on these results, we established a gene list corresponding to the non-redundant overexpressed genes in the FL context whatever the considered cell population. These 3405 up-regulated genes were postulated to be representative and specific of the FL supportive niche (Table S1). Metabolic and cell signaling pathways implicated in this FL niche were analyzed using gene-set enrichment analysis (GSEA). We selected the top 25 pathways according to the EnrichR combined scores and NCI Nature or Panther databases (Figure 2A,B, respectively). Interestingly, we could identify pathways related to the inflammation and the lymphocyte (and especially T cell) activation (Figure 2). T cell activation could also be evoked by highlighting the IL-6, IL-12, and IFN-γ pathways, and FoxO family signaling. Furthermore, besides lymphocyte-mediated signals, several strongly represented networks could be linked to stromal supportive niches and extracellular matrix, including integrin signals, Notch pathway, *n*-cadherin, or VEGF/VEGFR-mediated events, as well as TGF receptor, FGF, and EGF receptor signaling pathways.

**Figure 3:**
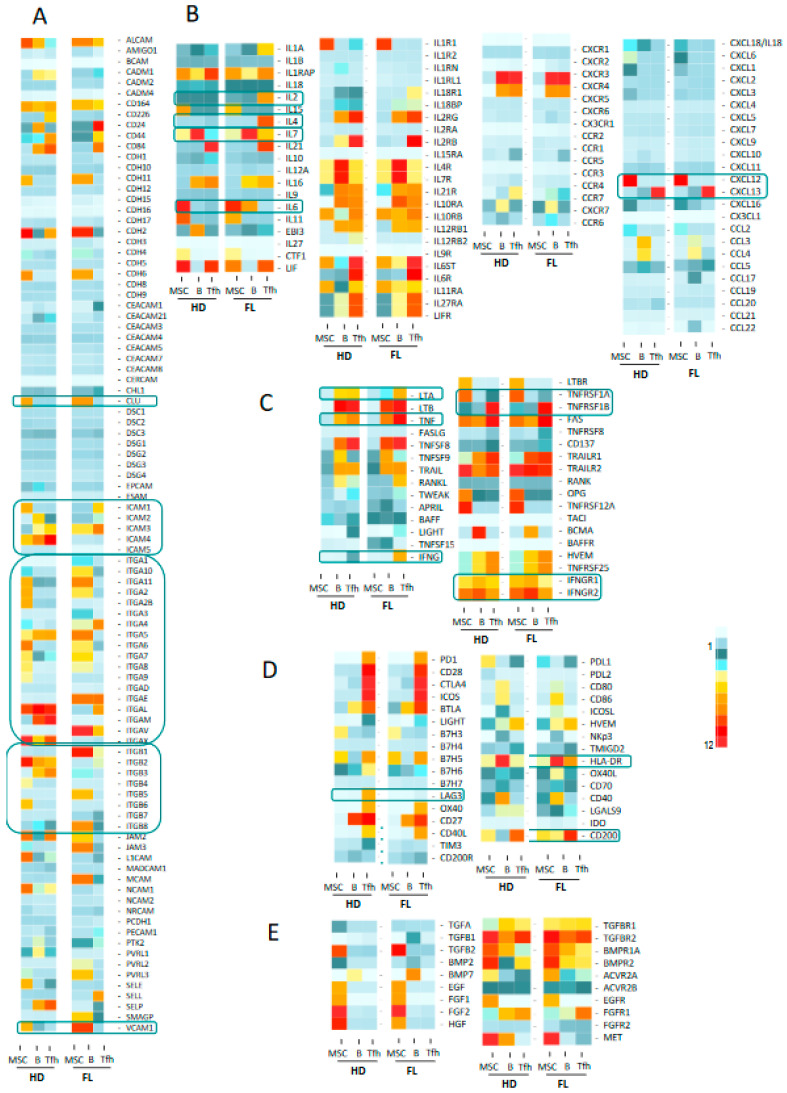
Heat map analysis of the expression of molecules and their relevant receptors by FL B cells, Tfh and mesenchymal stromal cells (MSC) in FL context compared to healthy donors (HD): adhesion molecules (**A**), cytokines and chemokines (**B**), TNF superfamily factors (**C**), immune checkpoints and co-stimulatory or inhibitory molecules (**D**) and TGF superfamily and other growth factors (**E**). Selected molecules correspond to non-redundant set of genes compiled from Refseq, NCBI and Genecard datasets. Data are means of normalized Affymetrix intensities for each molecule in each group.

We next questioned whether we could identify receptor–ligand pairs that could reflect the crosstalk between cell populations in the FL synapse (Figure 1A). To this end, we compiled information from Refseq, NCBI, and Genecard datasets, and we established a dataset of 258 genes encoding cytokines, chemokines, TNF family members, immune checkpoint molecules, growth factors, and their respective receptors, as well as a large list of adhesion molecules. We analyzed the three cell populations, and comparisons were drawn between nonmalignant and FL-derived populations (Figure 3 and Figure S2 for detailed expression of FL and HD B cell populations). This representation allowed us to finely identify and visualize potential cell communications at the FL synapse compared to the normal context. The analysis of the Ig superfamily members found an extinction of *ICAM1* and *ICAM4* expressions in FL-derived cell populations compared to normal counterparts, whereas *ICAM3* was clearly up-regulated (Figure 3A). Similarly, *ITGA5*, *ITGA10*, and *ITGAE* were overexpressed in FL B cells and FL-BM-MSC, whereas FL-Tfh up-regulated *ITGAE*. The expression of *VCAM1* was similarly up-regulated in FL-derived B and stromal cells, while the expression of clusterin *(CLU)*, in addition to its production by MSC, was up-regulated in tumor B cells. Taken as a whole, these findings suggest that the FL microenvironment is sustained by modifications of adhesion molecule expression, which could impact cell migration and cellular interactions. Interestingly, while the majority of the cytokine ligands/receptors were similarly expressed in FL and normal contexts, some cytokines were up-regulated in BFL cells, including *IL-6* and *IL-2*, or FL-Tfh, including *IL4* and *IL-7*, whereas their respective receptors were unmodified. Interestingly, FL-Tfh also overexpressed *IFNG.* Lastly, we noticed in FL that both B cells and Tfh up-regulated the expression of *CD200*, creating, together with its moderate expression by stromal cells, a CD200-rich milieu in the FL tissues. CD200 is known as a regulator of myeloid cell activity [20], opening a new gateway to other players in the FL niche.

### 2.2. Activation of Tfh Cells in FL Tumor

**Figure 4:**
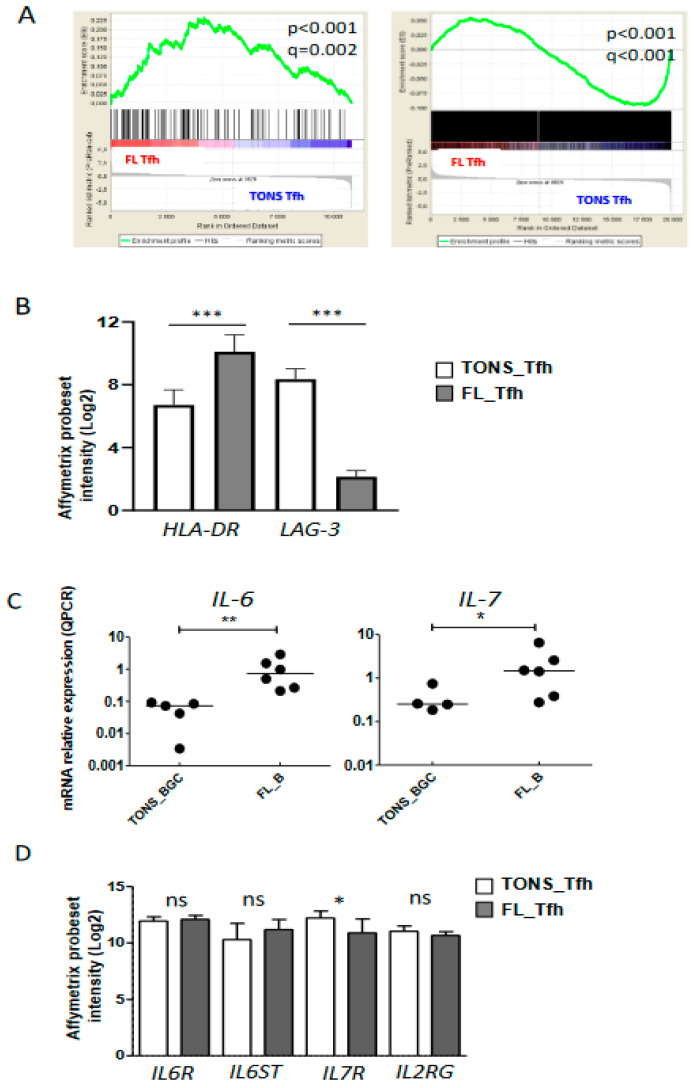
Tfh activation in FL tumor. (**A**) Plots from GSEA (gene-set enrichment analysis) analyses comparing Tfh from FL (FL Tfh) or TONS (non-malignant tonsils; TONS Tfh) based on an activated T signature (M3319 MSigDB geneset in left panel and activated Tfh signature obtained by Tfh stimulation with anti-CD3/CD28 mAbs (see method) in right panel). Nominal *p*-value (*p*), and FDR (q) are given on the plot. FL Tfh are given on the left (red) of the plot and normal TONS_Tfh on the right (blue). (**B**) *HLA-DR* and *LAG-3* mRNA expression on FL_Tfh or TONS_Tfh. (**C**) *IL-6* (left panel) and *IL-7* (right panel) mRNA expression by qRT-PCR in B lymphocytes isolated from reactive tonsils (TONS_BGC) (*n* = 5) or FL tumors (FL_B) (*n* = 6). The arbitrary value of 1 was assigned to a pool of five whole tonsil cells. (**D**) Gene expression of *IL-6R* and *IL-7* subunits in normal Tfh (TONS_Tfh) or derived from FL (FL_Tfh). Bars: mean. ns non-significant, * *p* < 0.05, ** *p* < 0.01, *** *p* < 0.001.

We previously demonstrated that the FL microenvironment is enriched in Tfh cells [17] supporting malignant B cell growth [18,21]. As discussed previously, our current gene-expression analysis suggested an activation of FL-Tfh (Figure 2) and confirmed overexpression of *IL-4*, *IFNG*, *TNFA*, and *IL-2* (Figure 3B,C). To further characterize FL-Tfh features, we tested the immunological signatures of MSigDB collections using GSEA and identified in FL-Tfh a significant enrichment for transcription factors belonging to a 193-gene signature specific to activated CD4^+^ T cells (M3319 geneset [22]) (Figure 4A, left panel).

We then built our own activated-Tfh signature by comparing the transcriptome of tonsil-purified Tfh (*n* = 7) previously stimulated or not by anti-CD3/CD28 monoclonal antibodies. We found 2824 differently expressed genes (*p* < 0.05, FC < 2) corresponding to 3665 PS and constituting a Tfh-specific activated signature. Through GSEA analysis, we showed that FL-Tfh were significantly enriched for this signature (*p* < 0.001) compared to their normal counterpart (Figure 4A). In agreement with this activated status, FL-Tfh expressed higher levels of *HLA-DR* (*p* < 0.001) and lower levels of the exhaustion marker LAG3 (*p* < 0.001) than tonsil-derived Tfh (Figure 4B). The differentiation and activation of Tfh follow a complex process, in which IL-6 and IL-7 are mandatory [23], IL-6 by inducing Tfh generation and differentiation in the GC [24,25] and IL-7 by fostering their differentiation and initiating memory program [26]. Our analysis showed that FL B cells overexpressed these two cytokines (Figure 4C). Both *IL6R* and *IL6ST/gp130*, the two IL-6 receptor subunits, were highly expressed by both normal and FL-Tfh, whereas *IL7R* expression was slightly down-regulated in FL-Tfh (Figure 4D). Among genes encoding costimulatory molecules, *CD70* and *CD80* were similarly expressed by FL and normal GC B cells, while *CD86* was significantly overexpressed in tumor cells (*p* = 0.004) (Figure S3), and could contribute to the activation of Tfh after binding to CD28. In addition, FL B cells increased their expression of *ICOSL* compared to normal GC B cells (*p* = 0.025) (Figure S3), and could thus participate in the recruitment and the activation of ICOS^pos^ Tfh [23,27]. Molecular data were furthermore confirmed in FL B cells using RNA sequencing on an independent cohort of FL patients and healthy donors (Supplementary Materials Figure S4, unpublished data). Taken as a whole, our study found at least three pathways, i.e., IL-6, CD86, and ICOSL, by which tumor B cells can contribute to Tfh activation and induce a potent microenvironment sustaining, in turn, FL cell growth.

### 2.3. Impaired Response of FL B Cells to IFN-γ

**Figure 5:**
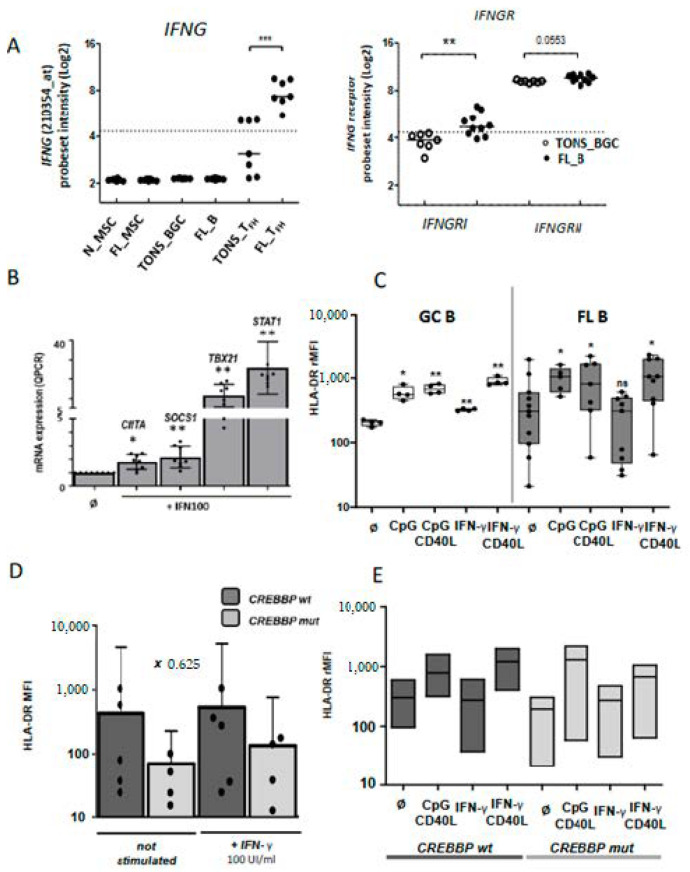
IFN-γ pathway in malignant B cells: (**A**) Up-regulation of *IFNG* (left panel) and *IFNGR* (right panel) mRNA expression on normal and FL subpopulations. (**B**) mRNA expression by qRT-PCR of IFN-γ-target genes (*CIITA, SOCS1, TBX21* and *STAT1*) in purified FL B cells stimulated or not (Ǿ) by IFN-γ 100 UI/mL (IFN100) for 24 h. (**C**) HLA-DR cell-surface expression on normal GC B cells (left panel) or FL B cells (right panel) cultivated for 24 h with CPG or IFN-γ, with or without CD40L stimulation, and according to *CREBBP* mutation. (**D** and **E**) rMFI: ratio of mean fluorescence intensity, wt: wild type, mut: mutation; Bars: mean. ns non-significant, * *p* < 0.05, ** *p* < 0.01, *** *p* < 0.001 compared to normal (**A**) or non-stimulated conditions (**B** to **E**).

We next evaluated the functional effects on FL B cells of molecules produced by Tfh. We previously demonstrated the supportive role of Tfh on FL B cells survival through CD40L expression and IL-4 secretion [18,21]. Herein, the above transcriptome analysis identified IFN-γ in the top five pathways involved in the FL synapse with an increased expression of *IFNG* by FL-Tfh, and this finding was confirmed on an independent series of FL (Figure 3D and Figure 5A left). Interestingly, *IFNGRI*, a gene encoding the ligand-binding alpha chain of IFN receptor, was also increased in FL-B cells compared to tonsil counterparts (Figure 5A, right, and Figure S4). We tested functionally purified FL B cells in vitro and left them overnight in culture in the presence or not of recombinant IFN-γ. At a first glance, we did not detect any differences between these two conditions in terms of cell proliferation and viability, even when CD40L and IL-4 were added to the culture (*n* = 4). Quantitative RT-PCR assays showed a dose-dependent increase of IFN-γ-regulated *CIITA, SOCS1, TBX21,* and *STAT1* (Figure 5B). IFN-γ is well known to induce molecules of the class II major histocompatibility complex (MHC) and could thus contribute to the immune response, a potentially deleterious process for the development of lymphoma. Unlike normal GC B cells, FL B cells were unable to increase their cell-surface expression of HLA-DR in response to IFN-γ, whereas enhanced HLA-DR expression was observed after CpG and/or CD40L exposure (Figure 5C). We thus demonstrated a specific defect on the IFN-γ-driven HLA-DR expression in FL B cells. The previous report of Green et al. connected the down-regulation of *HLA-DR* gene expression in FL B cells to a gene-silencing mechanism due to loss-of-function mutations of CREBBP [28]. In our study, all explored FL cases presented an altered IFN-γ-response in term of HLA-DR expression whatever the genotypic status of *CREEBP* (Figure 5D,E).

### 2.4. The FL Microenvironment Improves the Adhesion of B Cells to Stromal Cells

**Figure 6:**
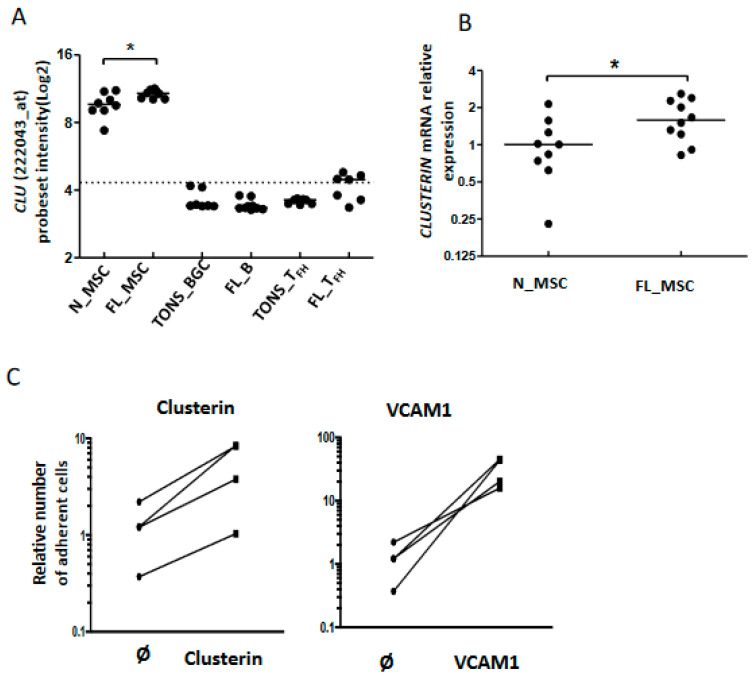
Adhesion of B cells to stromal cells via clusterin expression. Clusterin mRNA expression by stromal cells and up-regulation in the FL context evaluated by Affymetrix probeset intensity (**A**) and confirmed by qRT-PCR (**B**), * *p* < 0.05; (**C**): CFSE-labeled FL-B cells were incubated for 2 h in wells pre-coated with or without (Ø) clusterin (left), or VCAM1-Fc chimera protein as a control (right). The number of adherent cells was quantified using a fluorescence reader. The number of adherent naive B cells in wells without any coated protein was assigned as 1.

As described above, our data highlighted modifications among cell adhesion molecules, including *CLU,* at the interface between FL B cells and stromal cells (Figure 3A). Clusterin (also known as apolipoprotein J or ApoJ) is a molecule secreted following the stress response with a wide variety of effects depending on the cellular context [29] and which accumulates in lymphoid stromal cells [30]. Interestingly, MSC from FL-invaded BM showed a weak but significant increase of *CLU* gene expression (Figure 6A,B). This could be related to the commitment of FL BM stromal cells towards a lymphoid stromal cell differentiation [19]. Stimulation of FL B cells with clusterin for 4 days did not affect their viability or proliferation (data not shown). We next evaluated cell adhesion of FL B cells on clusterin, using VCAM1 as a positive control. We observed an increase of B cell adhesion in the presence of clusterin (Figure 6C). This result suggests that the production of clusterin by FL stromal cells improves the cell interactions within the microenvironment and contributes to creating a supportive stromal cell niche.

### 2.5. Enhanced CD200 Expression in FL Sustains a Tolerogenic Niche

**Figure 7:**
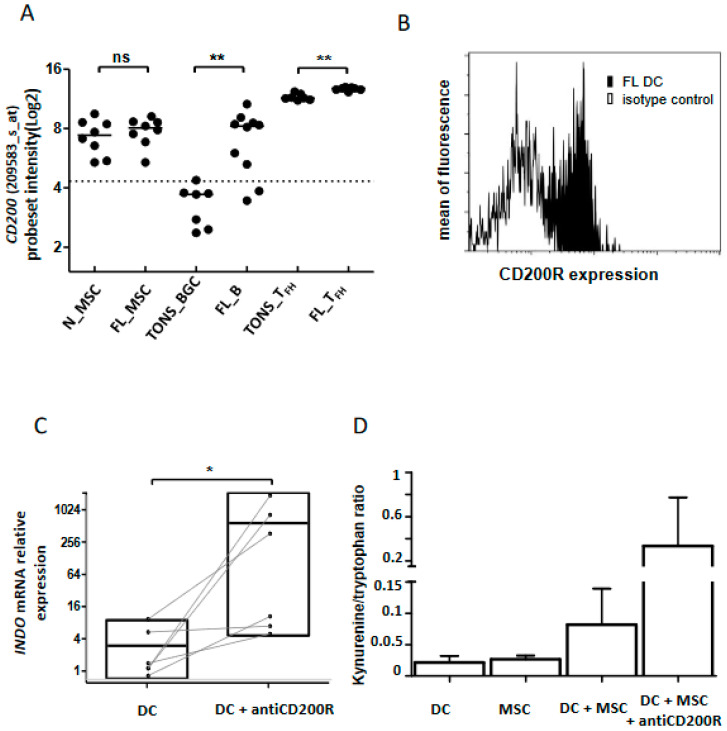
CD200 in FL tolerogenic niche. (**A**) *CD200* mRNA expression analyzed by Affymetrix probeset intensity in stromal cells from non-tumoral (*n*_STRO) or FL (FL_STRO) contexts, B lymphocytes from tonsil (TONS_BGC) or FL lymph node (FL_B) and Tfh lymphocytes from tonsil (TONS_Tfh) or FL lymph node (FL Tfh). ns: non-significant, ** *p* < 0.01; (**B**) Flow cytometry expression of CD200R on dendritic cells (DC) from FL lymph nodes (black histogram) compared to isotype control (white histogram). DC were phenotypically defined as CD3^neg^CD19^neg^CD335^neg^CD11c^pos^HLA-DR^pos^CD14^neg^ viable cells. One representative graph out of three experiments is shown; (**C**) *INDO* mRNA expression by qRT-PCR of immature DC with or without CD200R in vitro stimulation (20 µg/mL) for 2 days (*n* = 6), * *p* < 0.05; (**D**) IDO activity evaluated by kynurenine/tryptophan ratio on DC cultured or not with MSC and stimulated or not with anti-CD200R antibody (20 µg/mL) for 2 days.

Among the molecules up-regulated in the FL niche, *CD200* emerged in both B cells and Tfh (Figure 7A and Figure S4). It has been previously shown that binding of CD200 on its receptor, CD200R, induces an immunosuppressive/tolerogenic signal in the targeted cells [20,31]. Since none of the three explored cell populations expressed CD200R (Figure 3E), we speculated that this CD200-enriched milieu could impact myeloid cells and especially DC, leading to an immune suppressive effect. Indeed, the binding of CD200 to its receptor induces the production of IDO and IL-10 [32,33]. After validation by flow cytometry of CD200R expression on classical DC (cDC) issued from FL samples (Figure 7B and Figure S4), we performed in vitro stimulation of monocyte-derived DC by an agonist anti-CD200R antibody and evaluated *INDO* expression as well as the functional catalytic activity of IDO. Interestingly, we observed an induction of *INDO* expression (*n* = 6, *p* = 0.02) (Figure 7C) concomitantly with an up-regulation of the kynurenine/tryptophan ratio (Figure 7D) signaling the functionality of IDO. Moreover, the co-culture of DC with stromal cells synergized to trigger IDO activity in response to CD200R activation (Figure 7D), demonstrating that the FL niche is an CD200-enriched milieu with potential immunosuppressive functions.

## 3. Discussion

FL B cells need the support of immune and stromal cells, organizing together a protumoral microenvironment that sustains tumor B cell survival and growth. The FL microenvironment displays a preeminent amplification of CD4^pos^ T cells characterized by Tfh features but with a modified expression profile compared to their counterparts issued from reactive non-malignant LN [17,18]. The stromal compartment of the tumor is also modified, creating a permissive niche directly and indirectly supporting lymphoma cell growth [16,19].

**Figure 8:**
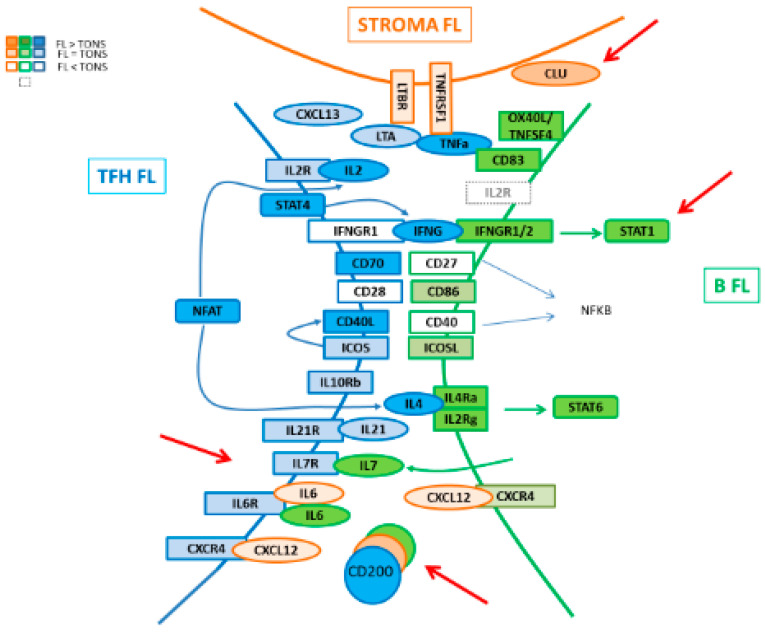
Schematic representation of interactions in FL niche.

In the present work, we addressed a description of the molecular pathways associated with the FL niche by exploring the GEP of the three major cell players, namely Tfh, MSC, and FL B cells, giving a global picture of the cell crosstalk between these compartments (Figure 8). In FL, Tfh present an activated signature which could enhance their capacity to promote lymphomagenesis through an increased expression of TNFα, LTA, IL-4 and CD40LG, associated with a maintained high production of CXCL13, ICOS, CTLA4, and CD200. These modifications of a Tfh-functional phenotype in FL could be in turn fostered by tumor cells through their increased expression of *IL-6* and *IL-7*, both implicated in T cell homeostasis, memory T cell generation [34,35,36], and Tfh activation [24,37]. Indeed, IL-6 triggers Tfh commitment through induction of the transcriptional repressor Bcl-6 [24], while IL-7 signaling represses Tfh-associated genes, including *BCL6* and *CXCR5,* and induces anti-apoptotic *BCL2* gene and glycerol channel aquaporin *Aqp9*, both of which are known to promote the long-term survival of memory cells [26]. Mc Donald et al. demonstrated that Tfh and central memory T (Tcm) cells share developmental pathways and can be co-initiated from a population of Th1 cells resulting in a Tfh/Tcm-like population. The IL-6 treatment of these IL6-R^+^ IL7-R^+^ Th1 cells results in a further increase of Tfh, whereas IL-7 treatment favors the initiation of the Tcm gene program. In our study, unlike the *IL6R* gene which maintained its expression in FL-derived Tfh, *IL7R* expression decreased, suggesting a potentially higher exposure of these cells to IL-6 which could promote a Tfh-memory cell differentiation [38].

Of interest, it has been shown in the inflammatory non-tumoral context that IL-6 supports the expansion of IFN-γ-secreting CD4^+^ T cells [39]. In FL, Tfh increase their expression of IFN-γ whereas FL B cells up-regulate IFNGR. However, FL B cells lost their ability to induce the expression of HLA-DR on the cell surface in response to IFN-γ, whatever the mutational status of *CREBBP,* whereas this induction is maintained in response to TLR9 and CD40L. Because HLA-DR functions in antigen presentation, it is likely that disabling cellular components of the immune system associated with tumor recognition and rejection could thwart immune-mediated death. Thus, impaired HLA-DR expression on FL B cells affects the antigen-presenting capability of these cells and thus participates in the control of immune surveillance.

Immune suppression occurs naturally via multiple mechanisms and tumors employ various mechanisms to evade immune surveillance. An immune inhibitory ligand/receptor pair that maintains immune quiescence is CD200/CD200R. The CD200 (OX-2 antigen) molecule is a type I immunoglobulin superfamily membrane glycoprotein, expressed in multiple cell types [20], whereas CD200 receptor (CD200R) is restricted to hematopoietic cells, mainly on the myeloid/monocyte lineage cells. CD200-CD200R engagement imparts an immunoregulatory signal leading to the suppression of a T-cell-mediated immune response [40,41], possibly with the induction of regulatory T cells and post-transplant tolerance [42,43]. CD200 expression was found up-regulated in several mature hematopoietic-related cancers [31,44] with a potential prognostic impact such as in multiple myeloma [45]. Interestingly, in our study, we pointed out the presence of CD200-enriched milieu in FL, and other partners such as monocytes [46] or DC [47] have already been described as contributors to the FL microenvironment by promoting tumor growth through the recruitment of accessory immune cells. Previous studies showed that a pro-inflammatory environment and IFN-γ were able to increase CD200 expression by BM-MSC [48]. Thus, the highlight of an increased IFN-γ expression by FL Tfh could explain the enhanced production of CD200 in the FL microenvironment, which provokes a CD200/CD200R engagement on myeloid cells initiating the expression of IDO activity leading to tryptophan catabolism in the FL microenvironment and T cell suppression. IDO activity might thus play an important role in regulating immune responses as a potent tool to help escape assault by the immune system, as described in DLBCL, where IDO expression was correlated with worse outcome after R-CHOP treatment [49].

Normal lymphocytes at different stages of maturation show different capacities to recirculate and to adhere to stromal cells. Cellular adhesion molecules are necessarily critical in these processes. Since malignant cells may retain some of the attributes of their normal counterparts, it is often postulated that they use the same molecules to achieve the same functional outcome as normal cells. However, studies usually focusing only on malignant cells neglect to explore other partners in the tumor microenvironment. In the present work, we jointly analyzed FL B cells, Tfh, and MSC in normal and tumor contexts, and this allowed us to highlight the remodeling of the Ig superfamily and integrin family molecules in the FL supportive niche, as we described extinction of some ICAM or integrin molecules, whereas others were up-regulated in the different partners. In accordance with an enhanced interaction between FL B cells and stromal cells, the up-regulation of the clusterin expression by stromal cells fits perfectly by promoting intense B cell adhesion. In addition, clusterin, which has been found to be induced by IFN-γ in Hodgkin’s lymphoma [50], is considered as an oxidative stress regulatory molecule preventing cell apoptosis [51], another potential role contributing to the FL niche development. Of note, the CD44 molecule is described to strongly interact with hyaluronan, a major component of the extracellular matrix and which plays a pivotal role in inflammation and cancer [52] as well as in locomotion and B cell migration on stromal cells and reticular fibers [53]. Interestingly, both IFN-γ and IL-4 have been shown to induce a reduction in CD44 cell-surface expression [54]. We here confirmed a weaker *CD44* gene expression in FL B cells compared to GC B cells [55], which could be thereby due to the IFN-γ- and IL-4-enriched microenvironment in FL, thus limiting the recirculation and migration of tumor cells by controlling their rolling adhesion on stromal cells, and favoring their accumulation in GC.

## 4. Materials and Methods

### 4.1. Patient Samples

Tissues and BM aspirates used for this study came from subjects recruited under written informed consent recovery according to the Principles of the Declaration of Helsinki and the French National Cancer Institute (INCa) ethic committee recommendations. They were collected, anonymized, and cryopreserved as viable cells in the local hematology biobank (CRB-Santé, CHU Rennes - French Minister Authorization DC-2016-2565) before diagnosis and/or staging evaluation. LN samples (*n* = 33) and BM aspirations (*n* = 8) were obtained from primarily diagnosed or non-treated FL patients. Normal B and T cells were obtained from non-malignant tonsils (TONS) (*n* = 14) collected from children undergoing routine tonsillectomy and normal BM aspirates (*n* = 8) were issued from patients undergoing cardiac surgery. All FL clones showed a predominantly CD10^pos^ follicular growth pattern classified into grade 1–2 (70%) or 3a (30%), according to the World Health Organization diagnostic criteria. Patients with grade 3b FL or with disease in relapse after treatment and transformed FL were excluded from this study.

### 4.2. Preparation of Highly Purified Cells

Fresh tissues of lymph nodes or tonsils were mechanically dissociated and flushed with syringe and needle. Cell suspensions were filtered, washed and subsequently sorted using combinations of monoclonal antibodies (mAbs) (Table S2A). Briefly, FL B cells and non-malignant tonsil GC B cells were sorted using FACSARIA (BD Biosciences) as CD20^hi^CD44^lo^CD38^pos^IgD^neg^CD138^neg^ cells for gene-expression profiling (FL LN, *n* = 17 and tonsils *n* = 14), and/or by magnetic sort using B cell isolation kit II (Miltenyi Biotech)) for culture experiments (*n* = 15). Tfh cells were obtained from CD4^pos^ T-cell-enriched fraction of FL LN (*n* = 7) or tonsils (*n* = 7) using the CD3^pos^CD4^pos^CXCR5^hi^ICOS^hi^CD25^neg^ definition as previously described [56]. Stromal cells were obtained after in vitro culture of adherent cells from healthy (*n* = 8) or FL (*n* = 8) BM samples, as previously reported [19].

### 4.3. Gene Expression Profiling

Total RNAs were extracted using AllPrep™ DNA/RNA Mini kit (Qiagen, Valencia, CA, USA) or with Qiazol (Qiagen) reagent, including DNAse treatment, as recommended by the manufacturer. RNA quality was evaluated by capillary electrophoresis using the Bioanalyzer 2100 (Agilent, Santa Clara, CA, USA). The gene expression profiles (GEP) were determined on Affymetrix Human Genome U133 Plus 2.0 microarrays containing 54,675 probe sets (PS). Hybridization and raw data of expression signal intensities extraction were processed by the CIT platform (www.cit.ligue-cancer.net). Data are available via the NCBI Gene Expression Omnibus (GSE85233 for B lymphocytes, GSE85229 for BM-MSC fractions and GSE66384 for Tfh populations). Raw data of all samples were first normalized together by the Robust Multichip Averaging algorithm using GC content (GC-RMA) and Log2 transformation with Partek^®^ Genomics Suite^TM^ software (Partek, St. Louis, MO, USA). Filtering was performed by exclusion of all PS with an intensity below the background threshold (assumed to log_2_ (20) in all samples) and/or without a gene symbol assignment. Principal component analysis and hierarchical clustering analyses (HCA) were achieved with R software. In parallel, pooled raw data of normal and tumor cell sample compartments (i.e., Tfh cells, B cells, and MSCs) were normalized and filtered as described above. Pairwise global gene expression comparison was performed using R. Probesets differentially expressed were then identified using the Limma moderated t-test [57] with false discovery rate (FDR) correction of *p*-values (FDR < 5%).

### 4.4. Tools for Affymetrix Dataset Analysis

Gene-set enrichment analysis (GSEA) [58] was performed through EnrichR method [59] and NCI Nature [60] or Panther [61] metabolic and cell signaling pathway databases. This approach combines gene function, ontology, pathways, and statistical analysis tools to rank enriched terms. Genes were explored through functional pathways ordered by statistical overrepresentation using combined scores (cScore) corresponding to the multiplication of the *p*-value (Fisher exact test, *p* < 0.05) and the z-score of the deviation from the expected rank.

### 4.5. Supplementary Method for GEP Analysis

Lists of known receptor–ligand pairs were elaborated using the NCBI database and Genecard datasets. Each selected molecule was associated with its ascension number, HGNC gene symbol and synonyms, and then linked to the Affymetrix database (Netaffx) in order to identify all corresponding PS. The PS intensities were selected in our normalized microarray dataset, and only one was retained according to successive filters: (1) expression by at least one sample in the dataset, (2) highest intensity in the dataset, and (3) highest standard deviation within compartments. The selected data were then adjusted by mean centering and intensity plots were visualized separately for FL and normal subsets using d3Heatmap R package.

### 4.6. Quantitative RT-PCR

RNAs were reverse transcribed into cDNAs using Superscript II and random hexamers (Invitrogen, Carlsbad, CA, USA). Quantitative RT-PCRs (qRT-PCR) were performed using the TaqMan Universal Master Mix and specific Taqman Gene Expression Assays from Applied Biosystems (Foster City, CA, USA) (Table S2B). *ABL* or *GAPDH* were determined as appropriate internal standard genes. Gene expression was measured using the ΔCT calculation method.

### 4.7. Flow Cytometry Analysis

Antibodies used for phenotyping are listed in Table S2A and appropriate isotype-matched mAbs were used as negative controls. Analyses were performed using a Gallios (Beckman Coulter) flow cytometer and data were analyzed using Kaluza software (Beckman Coulter). Cell death was checked using DAPI (Life Technologies) staining.

### 4.8. Primary B or T Lymphocyte Culture

Cultures were performed in complete medium consisting of RPMI 1640 (Invitrogen, Carlsbad, CA, USA) supplemented with FCS (Biowest, Nuaillé, France), antibiotics (Invitrogen). Tonsil-Tfh were cultured in vitro for 14 h with anti-CD3 and anti-CD28 antibodies (Sanquin, Amsterdam, The Nederlands) before RNA extraction for activated Tfh cell signature obtention. FL B cells were stimulated with or without gamma interferon (IFN-γ) (20 or 100 UI/mL, R&D Systems).

### 4.9. Dendritic Cell Production and Culture

Peripheral blood monocytes were obtained using CD14^pos^ Microbeads kit (Miltenyi Biotech) or by elutriation and were cultured for 5 days with GM-CSF (800 UI/mL, Cellgenix, Breisgau, Germany) and IL-4 (250 UI/mL, R&D Systems). Immature DC were then washed and put again in culture for 2 days with or without agonist anti-CD200R antibodies (20 µg/mL, R&D Systems) or isotype control. Co-cultures were performed in parallel with stromal cells (40,000 cells/cm^2^) and/or immature DC (1 × 10^6^ cells/mL) that were cultured for 5 days with GM-CSF (800 UI/mL) and IL-4 (250 UI/mL). CD200R stimulation was then added for 2 supplemental days of culture. Culture supernatants were collected by centrifugation, whereas cells were used for RNA extraction. In co-culture conditions, DC were separated from stromal cells using CD45^pos^CD105^neg^ gating strategy and FACSARIA cell sorting.

### 4.10. Indoleamine 2,3-Dioxygenase (IDO) Activity Analysis

IDO activity was evaluated on stromal cells and DC co-culture supernatants by measuring kynurenine concentration in culture supernatants by high performance liquid chromatography using 3-nitro-L-tyrosine as an internal standard. Kynurenine and 3-nitro-L-tyrosine were detected by UV absorption at 360 nm.

### 4.11. Adhesion Assay

Naïve B cells or primary FL B cells were isolated as previously described [18] with a purity greater than 95% of CD19^pos^ B cells expressing the appropriate malignant isotype light chain. Subsequently, FL B cells (*n* = 4) were labeled with 2 µM CFSE (Life Technologies) and 10^5^ FL B cells were seeded in each well of a pre-washed 96-well plate pre-coated with 5ug/mL of recombinant human VCAM1 Fc chimera protein (RD Systems) or 1.25ug/mL of recombinant human clusterin (RD Systems). Plates were then incubated 2 h at 37 °C. Thereafter, each well was washed once with PBS before quantification of residual fluorescence (excitation: 495nm; emission: 519 nm) using a Varioskan Flash Multimode reader (Thermo Scientific), which was directly correlated with the number of adherent cells. In order to quantify the number of adherent cells in each well, a titration curve was drawn using a standard range from 1.6 × 10^5^ to 781 cells per well by two-fold serial dilution, and by measuring the fluorescence in each well.

### 4.12. Statistical and Bioinformatic Analyses

Statistical analyses were performed with Prism software (GraphPad Software, La Jolla, CA, USA) using the Student’s t test or the Mann–Whitney nonparametric U test as appropriate.

## 5. Conclusions

Our study uncovers a global picture of the cell interactions within the FL microenvironment that contribute to the maintenance and development of a FL-specific tumor niche. In recent years, the application of genome-wide techniques has allowed identification of numerous genetic alterations in FL, which may impact interactions between the malignant B cells and the tumor microenvironment. It is therefore highly plausible that the FL niche includes the notion of tumor heterogeneity with molecular modifications depending on B cell genetic alterations, a hypothesis not addressed in the current study due to low number of cell samples

Our findings highlight the crucial role of bidirectional cell crosstalk to set up a supportive microenvironment for malignant FL cells that accumulate and escape immune surveillance before accumulating high genetic alterations leading to an aggressive lymphoma transformation. Targeting these cell interactions with specific drugs in the FL niche could represent an attractive option for future therapeutic strategies.

## Figures and Tables

**Table 1:** Characteristics of cohort for transcriptomic analysis.

	Non-Malignant Patient Derived Cells	FL-Derived Cells
B lymphocytes	7 tonsils from children	10 FL lymph nodes
Tfh lymphocytes	7 tonsils from children	7 FL lymph nodes
Mesenchymal stromal cells	8 HD bone marrow	8 FL bone marrow

## Supplementary Materials

The following are available online at https://www.mdpi.com/2072-6694/12/10/2865/s1, Figure S1: Hierarchical clustering the 7760 Affymetrix probesets (PS) with the higher intensities in each dataset; Figure S2: Heat map analysis of the expression of molecules and their relevant receptors by FL B cells compared to healthy donors (HD; Figure S3: *CD70, CD80*, *CD86* and *ICOSL* Affymetrix probeset intensities in FL tumor cells (FL_B) compared to tonsil germinal center B cells (TONS-BGC).; Figure S4: *IL6, IL-7, CD86*, *ICOSL*, *IFNGR* and *CD200* expression in sorted FL tumor B cells compared to tonsil germinal center B cells as assessed by RNA-seq (unpublished independent cohorts); Table S1: 3405 up-regulated genes in FL niche; Table S2: Antibodies used for flow cytometry and cell sorting, and Taqman gene expression assays used for RQ-PCR experiments
