# Supplementary material for: Integrative Analysis of Cell Crosstalk within Follicular Lymphoma Cell Niche: Towards a Definition of the FL Supportive Synapse
Source: Cancers (Basel). 2020 Oct 5;12(10):2865. doi: 10.3390/cancers12102865 (PMC7599549; doi:10.3390/cancers12102865)
Supplement: Supplementary file 1 [file cancers-12-02865-s001.zip › suppl fig-revised.pdf]

FigS1

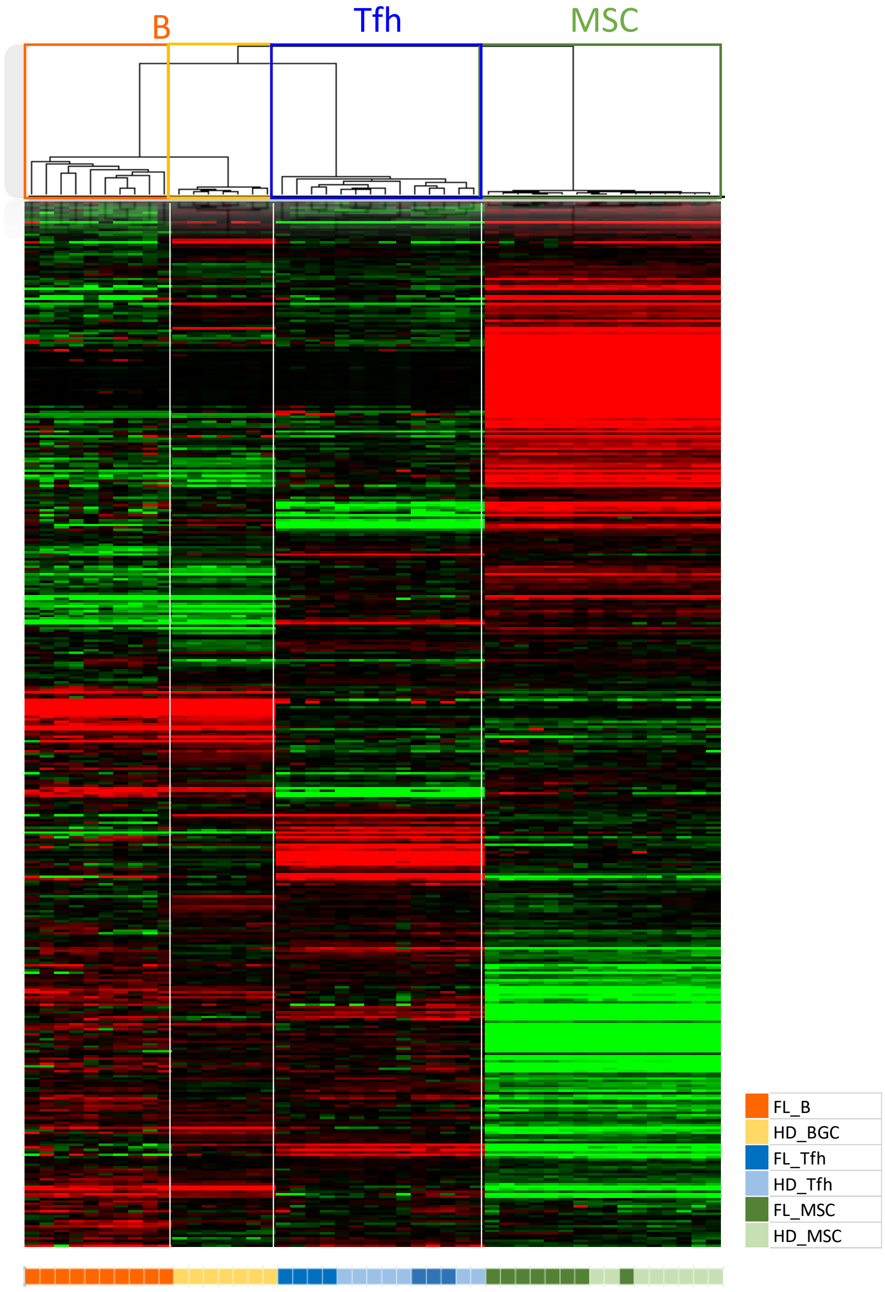

Figure S1: Hierarchical clustering the 7,760 Affymetrix probesets (PS) with the higher intensities in each dataset. Some of these PS are highly expressed in several subsets, so that here were selected 7,760 distinct non-redundant. FL : Follicular lymphoma, HD : Healthy donor, B : B lymphocytes, Tfh : follicular T helper lymphocytes, MSC : mesenchymal stromal cells.

Fig S2

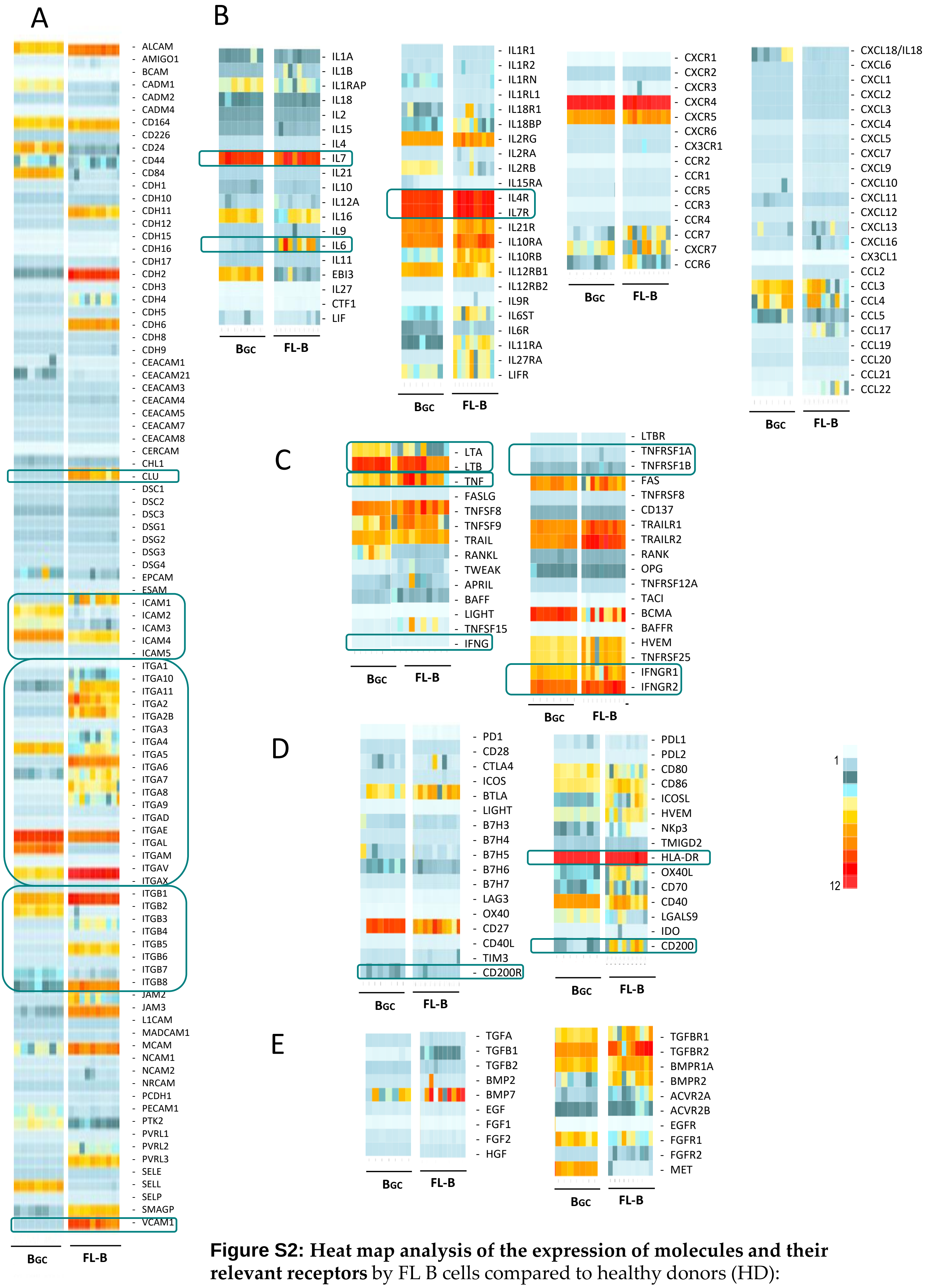

Figure S2: Heat map analysis of the expression of molecules and their relevant receptors by FL B cells compared to healthy donors (HD): Adhesion molecules (**A**), Cytokines and Chimiokines (**B**), TGF and TNF superfamilies' and others growth factors (**C**), Immune checkpoints and co-stimulatory or inhibitory molecules (**D**) and (**E**). Mean data are represented in Figure 3 with comparison to Tfh and mesenchymal stromal cells in FL and normal context.

FigS3

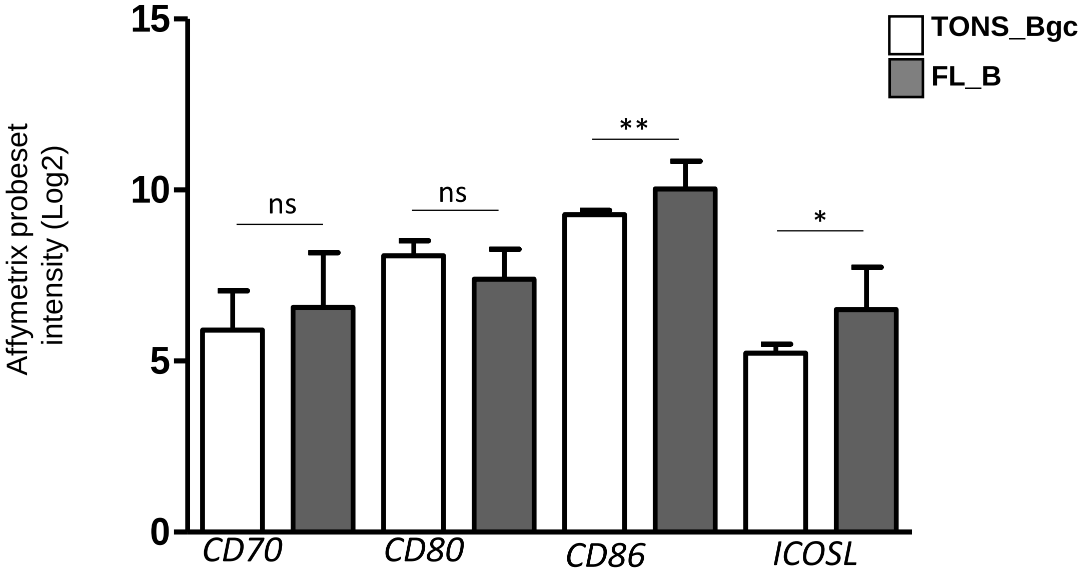

Figure S3: *CD70, CD80*, *CD86* and *ICOSL* Affymetrix probeset intensities in FL tumor cells (FL_B) compared to tonsil germinal center B cells (TONS-BGC).

FigS4

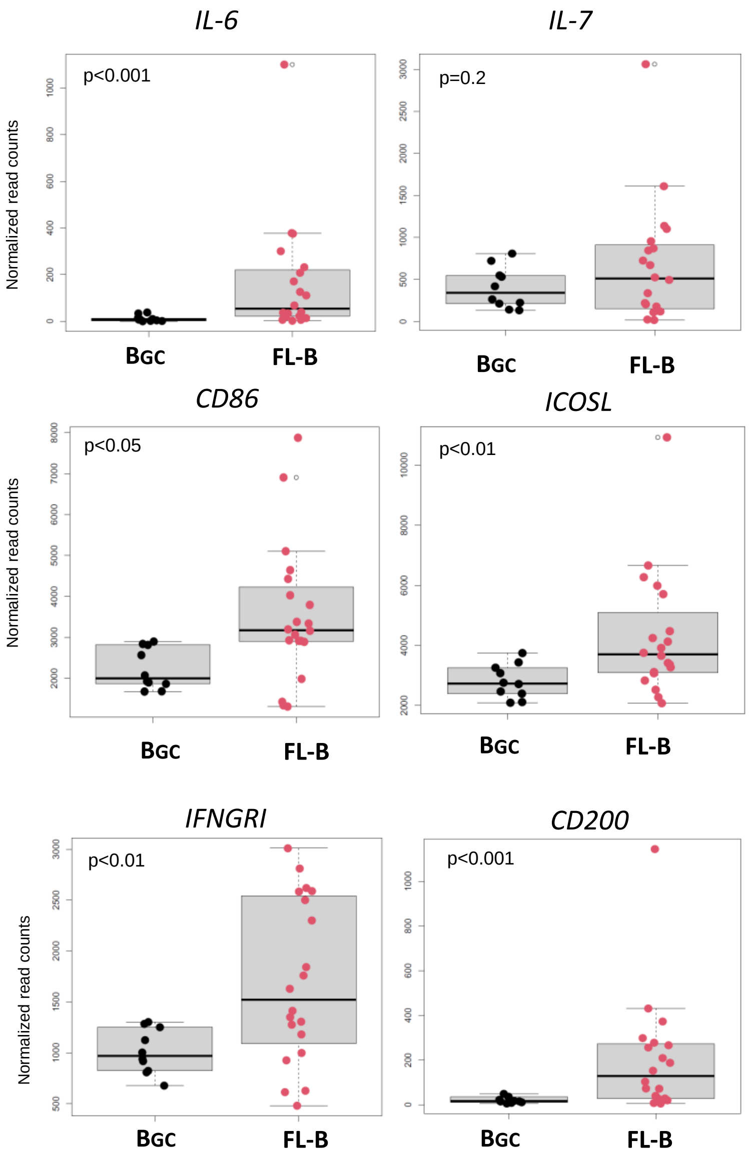

Figure S4: *IL6, IL-7, CD86,* ICOSL, *IFNGR and CD200* expression in sorted FL tumor B cells compared to tonsil germinal center B cells, as assessed by RNA-seq (unpublished data). 20 FL biopsies from patients and 5 tonsils from healthy donors were used to FACS-sort specific B cell subsets, RNA was extracted from sorted cells, QC qualified and sent for RNA-seq analysis to an external provider (Helixio, Clermont-Ferrand, France). Raw data were aligned on the hg19 reference genome and gene counts were used to perform a differential expression analysis with the R software and the DESeq2 package. Statistical difference was assessed by Wald's test followed by Benjamini-Hochberg correction for multiple testing.
