# Supplementary material for: Integrative Analysis of Cell Crosstalk within Follicular Lymphoma Cell Niche: Towards a Definition of the FL Supportive Synapse
Source: Cancers (Basel). 2020 Oct 5;12(10):2865. doi: 10.3390/cancers12102865 (PMC7599549; doi:10.3390/cancers12102865)
Supplement: Supplementary file 1 [file cancers-12-02865-s001.zip › Table S2A, B.docx]

**Suppl Table 2A:** Antibodies used for flow cytometry and cell sorting

| **Specificity** | **Fluorochrome** | **Supplier** | **Use** |
| --- | --- | --- | --- |
| **CD20** | ECD | Beckman coulter | B lymphocytes cell sorting by FACSARIA |
| **CD38** | PE | Beckman coulter |  |
| **CD44** | PC7 | eBiosciences |  |
| **CD138** | FITC | Beckman coulter |  |
| **IgD** | FITC | BD Biosciences |  |
| **CD4** | FITC | Beckman coulter | Tfh cell sorting  by FACSARIA |
| **CD25** | APC | Becton Dickinson |  |
| **CXCR5** | PE | R&D systems |  |
| **ICOS** | Biotin | eBiosciences |  |
| **Streptavidine** | PC7 | Beckman coulter |  |
| **CD200R** | Unconjugated | R&D | Flow cytometry mDC phenotyping |
| **CD3** | V450 | BD Biosciences |  |
| **CD11c** | A700 | BD Biosciences |  |
| **CD14** | PC7 | Beckman coulter |  |
| **CD19** | BV 421 | BD Biosciences |  |
| **CD123** | PerCP5.5 | BD Biosciences |  |
| **CD335** | BV 421 | Biolegend |  |
| **HLA-DR** | PE-CF594 | BD Biosciences |  |
| **Viable cells** | ZombieYellow | Biolegend |  |
| **CD45** | FITC | Beckman coulter | DC cell sorting by FACSARIA after in vitro culture with stroma cells |
| **CD105** | A647 | Biolegend |  |
| **DAPI** | - | Sigma | Exclusion of dead cells |

**Suppl Table 2B:** Taqman gene expression assays used for RQ-PCR experiments

| **Gene symbol** | **Gene names** | **Assay ID** |
| --- | --- | --- |
| **ABL** | c-abl oncogene 1 | hs001104728-m1 |
| **CIITA** | Class II major histocompatibility complex transactivator | hs 00172106-m1 |
| **CLU** | Clusterin | hs99999905-m1 |
| **GAPDH** | Glyceraldehyde-3-phosphate dehydrogenase | hs99999905-m1 |
| **IL6** | Interleukin 6 | hs00174131_m1 |
| **IL7** | Interleukin 7 | hs00174202-m1 |
| **INDO** | Indoleamine 2,3-dioxygenase 1 | hs00158032-m1 |
| **SOCS1** | Suppressor of cytokine signaling 1 | hs00705164_s1 |
| **STAT1** | Signal transducer and activator of transcription 1 | hs 01013996_m1 |
| **TBX21** | T-box 21 = TBET | hs00203436_m1 |
